# Early Systemic Microvascular Damage in Pigs with Atherogenic Diabetes Mellitus Coincides with Renal Angiopoietin Dysbalance

**DOI:** 10.1371/journal.pone.0121555

**Published:** 2015-04-24

**Authors:** Meriem Khairoun, Mieke van den Heuvel, Bernard M. van den Berg, Oana Sorop, Rients de Boer, Nienke S. van Ditzhuijzen, Ingeborg M. Bajema, Hans J. Baelde, Malu Zandbergen, Dirk J. Duncker, Ton J. Rabelink, Marlies E. J. Reinders, Wim J. van der Giessen, Joris I. Rotmans

**Affiliations:** 1 Department of Nephrology, Leiden University Medical Center, Leiden, The Netherlands; 2 Einthoven Laboratory for Experimental Vascular Research, Leiden University Medical Center, Leiden, The Netherlands; 3 Department of Pathology, Leiden University Medical Center, Leiden, The Netherlands; 4 Division of Experimental Cardiology, Department of Cardiology, Erasmus Medical Center Rotterdam, Rotterdam, The Netherlands; UCL Institute of Child Health, UNITED KINGDOM

## Abstract

**Background:**

Diabetes mellitus (DM) is associated with a range of microvascular complications including diabetic nephropathy (DN). Microvascular abnormalities in the kidneys are common histopathologic findings in DN, which represent one manifestation of ongoing systemic microvascular damage. Recently, sidestream dark-field (SDF) imaging has emerged as a noninvasive tool that enables one to visualize the microcirculation. In this study, we investigated whether changes in the systemic microvasculature induced by DM and an atherogenic diet correlated spatiotemporally with renal damage.

**Methods:**

Atherosclerotic lesion development was triggered in streptozotocin-induced DM pigs (140 mg/kg body weight) by administering an atherogenic diet for approximately 11 months. Fifteen months following induction of DM, microvascular morphology was visualized in control pigs (n = 7), non-diabetic pigs fed an atherogenic diet (ATH, n = 5), and DM pigs fed an atherogenic diet (DM+ATH, n = 5) using SDF imaging of oral mucosal tissue. Subsequently, kidneys were harvested from anethesized pigs and the expression levels of well-established markers for microvascular integrity, such as Angiopoietin-1 (Angpt1) and Angiopoietin-2 (Angpt2) were determined immunohistochemically, while endothelial cell (EC) abundance was determined by immunostaining for von Willebrand factor (vWF).

**Results:**

Our study revealed an increase in the capillary tortuosity index in DM+ATH pigs (2.31±0.17) as compared to the control groups (Controls 0.89±0.08 and ATH 1.55±0.11; p<0.05). Kidney biopsies showed marked glomerular lesions consisting of mesangial expansion and podocyte lesions. Furthermore, we observed a disturbed Angpt2/ Angpt1balance in the cortex of the kidney, as evidenced by increased expression of Angpt2 in DM+ATH pigs as compared to Control pigs (p<0.05).

**Conclusion:**

In the setting of DM, atherogenesis leads to the augmentation of mucosal capillary tortuosity, indicative of systemic microvascular damage. Concomitantly, a dysbalance in renal angiopoietins was correlated with the development of diabetic nephropathy. As such, our studies strongly suggest that defects in the systemic microvasculature mirror the accumulation of microvascular damage in the kidney.

## Introduction

Diabetes mellitus (DM) is associated with macrovascular and microvascular complications that lead to retinopathy, neuropathy and nephropathy. Diabetic nephropathy is the leading cause of chronic kidney disease (CKD) worldwide [1,2]. In this disease setting, the combination of endothelial dysfunction and an imbalanced angiogenic response play an important role in the development of microvascular complications. Recently, the angiopoietins have gained much attention as critical regulators of of diverse pathological angiogenic conditions, including vascular complications associated with diabetes. The tight control of Angiopoietin-1 (Angpt1) and Angiopoietin- 2 (Angpt2) levels is critical in minimizing microvascular derangements that are the direct result of negative interference of Angpt2 with Angpt1-mediated Tie-2 signaling. This in turn disturbs the expression levels of key angiogenic factors such as von Willebrand factor (vWF) and vascular endothelial growth factor (VEGF). The ensuing loss of EC-pericyte interactions is responsible for destabilization of the capillary network and the loss of microvascular integrity. [3,4, 5, 6].

Histopathological findings in patients with diabetic nephropathy (DN) include mesangial expansion, mesangial sclerosis and vascular lesions such as hyalinosis [7]. These renal abnormalities could potentially be indicative of the systemic microvascular damage [8]. However, the detection of such pathophysiologies in DN is complex, as it is asymptomatic in early stages, while at later stages the direct demonstration of renal injury requires renal biopsy material, which is an unattractive tool for screening purposes due to the invasive nature of the procedure. Therefore, methods geared towards the non-invasive monitoring for early signs of microvascular changes is of clinical importance in patients with DM. However, at present these tools do not exist. Sidestream dark-field (SDF) imaging is a relatively new, non-invasive tool that enables one to visualize the microcirculation without the use of fluorescent dyes [9]. Recently, we used this apparatus to assess labial mucosal capillary tortuosity and density in diabetic patients compared with healthy non-diabetic controls, studies that revealed increased capillary density and tortuosity in diabetic patients [10]. These studies focused on the effects of prolonged diabetes in patients (24 ± 14 years). However, at present there is limited data regarding how disturbance of the microvasculature in early diabetes correlates with renal damage.

In light of these considerations, we sought to investigate whether an atherogenic diet in the early stages of DM could accelerate microvascular disease, and could serve as a diagnostic tool for DM-induced renal damage. Using SDF imaging, we show that an atherogenic diet during the early stages of DM leads to microvascular abnormalities, and immunohistochemically confirm that these systemic effects are associated with renal endothelial dysfunction, as evidenced by a disturbed Angpt2/Angpt1 balance and microalbuminuria.

## Materials and Methods

### Animal experiments

The experimental protocol was performed in compliance with the ARRIVE guidelines on animal research [11]. Protocols describing the management, surgical procedures, and animal care were reviewed and approved by the ASG-Lelystad Animal Care and Use Committee (Lelystad, The Netherlands) and by the Institutional Review Board for animal experimentation of the Erasmus University Medical Center (Rotterdam, The Netherlands). Ten neutered male domestic pigs (Landrace x Yorkshire, T-line) with an age ~11 weeks and a body weight of ~30 kg entered the study. After one week of acclimatization, DM was induced in a subgroup of 5 pigs by slow intravenous injection of streptozotocin (STZ 140 mg/kg; Enzo Life Sciences, Raamsdonkveer, The Netherlands) as described previously [12–15]. Five non-diabetic pigs received placebo treatment. Three weeks after DM induction, all of the pigs were gradually introduced to an atherogenic diet (ATH), containing 25% of saturated fats and 1% of cholesterol [16]. The 5 diabetic pigs (DM+ATH) and 5 non-diabetic pigs (ATH) were subsequently followed for up to 15 months, during which similar growth patterns were observed in both groups by adjusting individual caloric intake. In the diabetic pigs, glucose and ketone levels in plasma and urine were regularly checked in the 15-month follow-up period. Insulin therapy was started only in instances where plasma ketones were detected. Following 12 months, 24h urine samples were obtained from all pigs, while microcirculatory imaging and plasma samples were obtained from anesthetized pigs (20mg/kg ketamine + 1 mg/kg midazolam+ 1 mg atropine, i.m. and 12mg/kg thiopental, i.v.) at 14 and 15 months of follow up, respectively. At 15 months, the pigs were sacrificed by intravenous injection of an overdose of pentobarbital via the jugular vein catheter at 15 months, at which point plasma samples were obtained and the kidneys were harvested for histological examination. Invasive imaging analyses prompted us to choose this time frame as after 12 months of follow up more complex atherosclerotic lesions were present in the coronary arteries of the studied pigs [23].

In addition, we also studied a separate control group (Controls) of 7 female domestic pigs (Landrace) at the age of 5 months with a body weight of ~78 kg, who were fed a standard diet for growing pigs.

### Microcirculatory imaging and analysis of SDF measurements

SDF microscanning (MicroVision Medical Inc., Wallingford, PA, U.S.A) and analysis of images was performed as described earlier with minor modifications [10,16]. Anaesthetized pigs were imaged at 14 months of study duration. All pigs were studied during standardized conditions of general anesthesia by a trained observer.

### Laboratory assessment

Serum Angpt1 and Angpt2 concentrations were measured after 12 months by enzyme-linked immunosorbent assay (ELISA) (Bioconnect, Huissen, The Netherlands and Antibodies-online, Atlanta, USA).

At 12 months, protein and creatinine levels were measured in 24h urine samples. To calculate the urinary albumin-creatinine ratio, we measured albumin excretion by ELISA (ITK diagnostics, Uithoorn, The Netherlands), and urinary creatinine concentration was measured by standard laboratory methods. Both the plasma and urinary creatinine levels were measured as previously described.

Plasma samples collected at 15 months were used to measure concentrations of glucose, total cholesterol, triglycerides and low and high-density lipoproteins (LDL and HDL) and creatinine by standardized methods at the clinical chemical laboratory of the Erasmus Medical Center (Rotterdam, The Netherlands). In the absence of an established mathematical formula to estimate creatinine clearance in pigs, we expressed creatinine levels in μmol/L/kg to adjust for body weight. Blood collection tubes were centrifuged for 10 minutes at 3000 rpm after which serum was stored in microcentrifuge tubes at -20°C until required for analysis.

### Histological analysis of the kidney

Immunohistochemical analyses of snap-frozen porcine kidney cortex sections (4 μm) were air-dried and acetone fixed as previously described [17]. Monoclonal antibodies utilized were directed against mouse anti-human Angpt1 (clone 171718; R&D Systems, Abingdon, UK), mouse anti-human VEGF (sc-7269 Santa Cruz Biotechnology, Huissen, the Netherlands) and mouse anti-human plasminogen activator inhibitor-1 (clone HD-PAI-1 14.1) (American Diagnostica, Pfungstadt, Germany). Polyclonal antibodies were used directed against goat anti-mouse Angpt2 (clone F18; sc-7017; Santa Cruz Biotechnology, Huissen, the Netherlands) and rabbit anti-human vWF (Codenr A0082; Dako, Glostrum, Germany). Primary antibody binding was detected using the following isotype-matching secondary horseradish peroxidase (HRP)-labeled antibodies; goat anti-mouse IgG (Angpt1), rabbit anti-goat IgG (Angpt2), anti-mouse Envision K4007 (VEGF) or goat anti-rabbit IgG (vWF) (all purchased from Dako, Glostrum, Germany). Quantification of immunohistochemistry was performed in a blinded manner by assessing 10 consecutive high power fields (magnification, ×200) using Image J software. Glomerular and large vessels were excluded from analysis. For VEGF-A quantification, 15 consecutive glomeruli per subject were selected and the percentage of VEGF-A positive area was calculated using Image J.

Data are presented in terms of the Angpt2/Angpt1 ratio as previously described[18,19]. Immuofluorescent double stainings were performed for desmin (pericyte marker; mouse anti-pig clone DE-B-5; Millipore, Amsterdam, the Netherlands)/Angpt1 (goat anti-human; clone N-18; sc-6319; Santa Cruz Biotechnology, Huissen, the Netherlands) and vWF/Angpt2. Secondary antibodies were Alexa-488 labeled donkey anti- rabbit (vWF) and alexa-546 labelled rabbit anti-mouse (desmin), Furthermore, HRP labelled rabbit anti-goat (Angpt1) and donkey anti-goat (Angpt2) were used. Nuclei were visualized using Hoechst (Molecular Probes, Leiden, the Netherlands). Photomicrographs were made using a fluorescence microscope (DM5500B; Leica, Rijswijk, the Netherlands).

Next, we performed periodic acid-Schiff (PAS) staining on paraffin sections of renal cortex for the evaluation of histopathological changes and morphometric analysis of the glomeruli. In a blinded fashion, 25 consecutive glomeruli were selected from both superficial and deep cortex and the mesangial expansion index was scored. Subsequently, we determined the extent of increase in mesangial matrix (defined as mesangial area) by quantitating PAS-positive and nuclei-free areas in the mesangium using Image J [20,21]. Evaluation of histopathological lesions was performed by a pathologist who was blinded to the code of the sections.

### Electron microscopy

Kidney tissues for electron microscopy were processed as described previously [22]. In typical cases from the ATH and DM+ATH groups, the glomerular basement membrane (GBM) width was measured.

### RNA isolation and Real-Time PCR

To analyze renal Angpt1 and Angpt2 mRNA levels, RT-PCR was performed as described previously [23]. Expression was normalized against β-actin mRNA levels. RT-PCR analysis for biological replicates was performed in duplicate. The primer sequences were as follows: β-actin (forward 5’-ATCGTGCGGGACATCAAGGA-3’ and reverse 5’-AGGAAGGAGGGCTGG-AAGAG-3’), Angpt1 (forward 5’-AGGAGCAAGTTTTGCGAGAG-3’ and reverse 5’-CTCA-GAGCGTTTGTGTTGT-3’) and Angpt2 (forward 5’-AAAGTTGCTGCAGGGAA-AGA-3’ and reverse 5’-TCACAGCTCAGAGCGAAGAA-3’).

### Statistical analysis

All data are presented as mean ± standard error of the mean (SEM). Differences between experimental groups were analyzed using one way-analysis of variance followed by post-hoc testing with an unpaired Student’s t-test. Analyses were performed with GraphPad software (GraphPad Software Inc., San Diego, CA, U.S.A.). P-values less than 0.05 (two-tailed) were considered statistically significant.

## Results

### Model characteristics

Initial plasma profiling of DM+ATH and ATH pigs revealed that DM significantly influenced glucose levels (17.64 ± 4.54 mmol/L vs. 5.12±0.42 mmol/L; p<0.05) (Table 1) at 15 months. Total cholesterol, triglycerides, HDL and LDL were not significantly affected by the induction of DM in pigs. Two pigs were administered insulin during the study period due to elevated plasma ketone levels. The mean body weight at the time of microvascular imaging was comparable between DM+ATH (96±2.8kg) and ATH group (93±0.4kg, p>0.05). At the time of sacrifice, substantial coronary lesions as well as generalised atherosclerosis for example in the aorta were observed in both DM+ATH and ATH groups, as described previously [24].

**Table 1:** Model characteristics at 15 months of follow up.

	Controls (N = 7)	ATH (N = 5)	DM+ATH (N = 5)
**Glucose (mmol/l)**	4.26±0.29	5.12±0.42	17.64±4.54* ^#^
**Triglycerides (mmol/l)**	0.18±0.02	0.64±0.2	1.26±0.50*
**Total Cholesterol (mmol/l)**	1.50±0.05	18.10±2.66*	16.82±1.72*
**LDL (mmol/l)**	0.76±0.29	14.87±2.45*	13.63±1.3*
**HDL (mmol/l)**	0.60±0.03	5.70±0.35*	5.12±0.40*

All data are mean ±SEM. ATH: pigs on atherogenic diet. DM+ATH: pigs with diabetes mellitus on atherogenic diet. Controls: healthy control pigs on a standard diet. LDL: Low density lipoprotein. HDL: High density lipoprotein.

* P<0.05 compared with Controls.

#P<0.05 compared with ATH pigs.

### Early atherogenic diabetes mellitus leads to systemic microvascular damage

**Fig 1:**
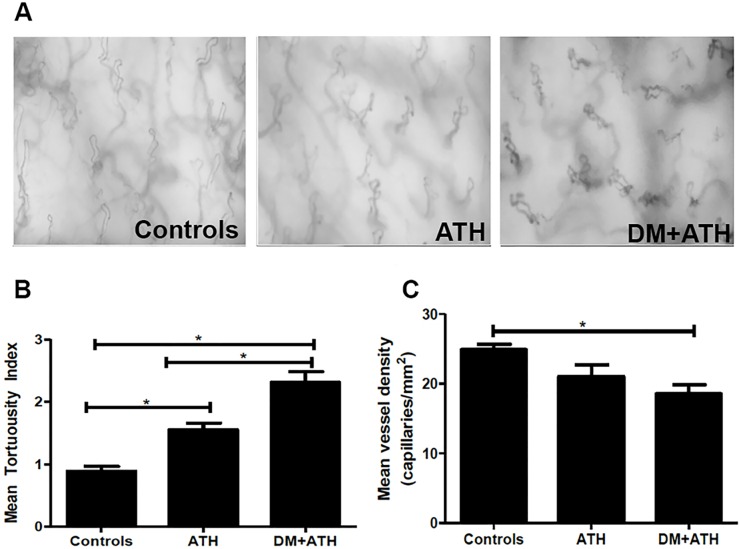
Early atherogenic DM leads to increased capillary tortuosity. A. Sidestream darkfield images of the oral mucosa visualizing the microvascular capillaries of a representative pig in the Controls, ATH and DM+ATH group. B. Mean tortuosity index of microvascular capillaries in the Controls (n = 7), ATH (n = 5) and DM+ATH pigs (n = 5). C. Mean vessel density (capillaries/mm^2^) in Controls (n = 7), ATH (n = 5) and DM+ATH (n = 5) pigs. Data shown are mean±SEM. *P<0.05 compared to Controls or ATH pigs. Mean vessel density (capillaries/mm^2^) was calculated by evaluation of number of vessels per selected microcirculatory image. Subsequently, tortuosity of capillary loops was assessed according to a validated scoring system (0: no twists to 4: four or more twists) and the average of assessed capillary tortuosity was used to calculate mean tortuosity index per patient.

The microvascular morphology of the oral mucosal tissue in DM+ATH pigs was significantly disturbed as compared with the Controls (Fig 1A). Indeed, the capillary tortuosity index was significantly increased in DM+ATH pigs (2.3±0.2) as compared with ATH pigs (1.6±0.1, p<0.01; Fig 1B). Furthermore, capillary density was significant lower in the DM+ATH (18.6 ±1.3 capillaries/mm^2^) pigs as compared with the Controls (24.9 ±0.8 capillaries/mm^2^, p<0.01; Fig 1C).

### Renal damage after induction of atherogenic DM in pigs

Plasma creatinine levels were elevated in ATH pigs (2.12±0.03 μmol/L/kg) as compared with DM+ATH pigs (1.60±0.13 μmol/L/kg, p<0.01) group. However, urinary albumin/creatinine ratio was not found to differ between the DM+ATH group (0.045±0.0182 mg/mmol) and ATH pigs (0.002±0.0007 mg/mmol, p>0.05).

**Fig 2:**
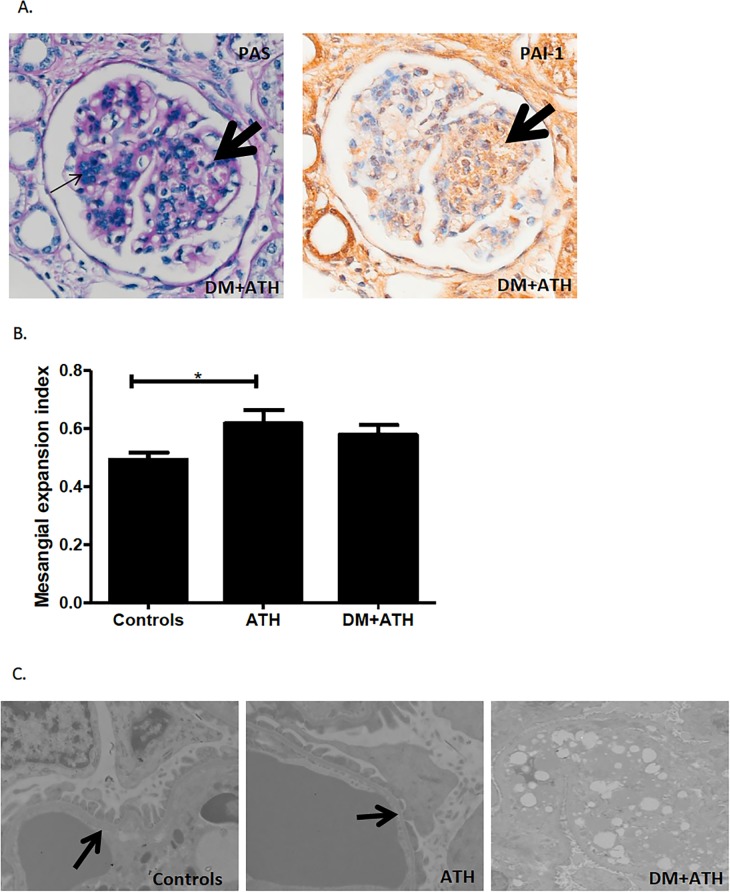
Early stages of atherogenic DM leads to renal damage. A: Representative illustration of PAS stained glomeruli from a DM+ATH pig, showing mesangial proliferation and matrix expansion with capillary loops lying around the mesangium as a corona, reminiscent of a beginning Kimmelstiel-Wilson nodule (left panel; thin black arrow). Dilated capillary loops with red cell fragments show intense PAI-1 staining on consecutive slides (right panel; thick black arrow). B: Mesangial expansion index in Controls (n = 7), ATH (n = 5) and DM+ATH (n = 5) pigs. C. Electron microscopy images illustrating a normal GBM architecture (left panel; thick arrow) of the Controls pig. In ATH, there is slight effacement of the podocyte pedicles (middle panel; thick arrow). In DM+ATH, marked lipid deposits were found (right panel). Data are shown as mean ± SEM. *P<0.05 compared to Controls or ATH pigs. Original magnification of A: x400 and C: x8000.

PAS-stained renal sections revealed marked tubular changes with foamy cytoplasm and hyalin droplets in all DM+ATH pigs and there were glomerular lesions consisting of mesangial expansion and podocyte lesions, also with foamy cytoplasm and hyaline droplets in four DM+ATH pigs (Fig 2A). In contrast, but one ATH pig displayed similar lesions (Fig 2A). Some glomeruli were found to have dilated capillaries containing numerous red blood cells, a phenomenon that is highly reminiscent of microangiopathic injury lesions that have been described to be the result of fibrinolytic/proteolytic activation system in combination with increased PAI-1 staining [25]. In keeping with this notion, we observed both co-localization and increased PAI-1 staining in the herein described lesions (Fig 2A).

Next, we determined the mesangial expansion index in renal cross-sections. These studies showed significantly higher scores in the ATH pigs (0.62±0.05, p = 0.03) and a trend towards increased scores in the DM+ATH (0.58±0.05,p = 0.066) compared with the Controls (0.49±0.03; Fig 2B). Interestingly, the width of the GBM increased from 215 nM in a Controls animal to 252 and 279 nM in cases with mild and severe DN of the DM+ATH group, respectively. Moreover, lipid deposits were observed in kidneys of mild and severe DN pigs (Fig 2C).

### Diabetes and atherogenic diet induces Angpt2/Angpt1dysbalance in pigs

Serum Angpt-1 levels in the DM+ATH pigs were increased as compared to control pigs (26035±1228 pg/ml versus 18061±1228 pg/ml, p<0.01). There were no significant differences in serum Ang-1 levels between DM+ATH and ATH pigs (19832±1166 pg/ml, p>0.05). Angpt-2 levels were not detectable in the circulation of the different groups.

**Fig 3:**
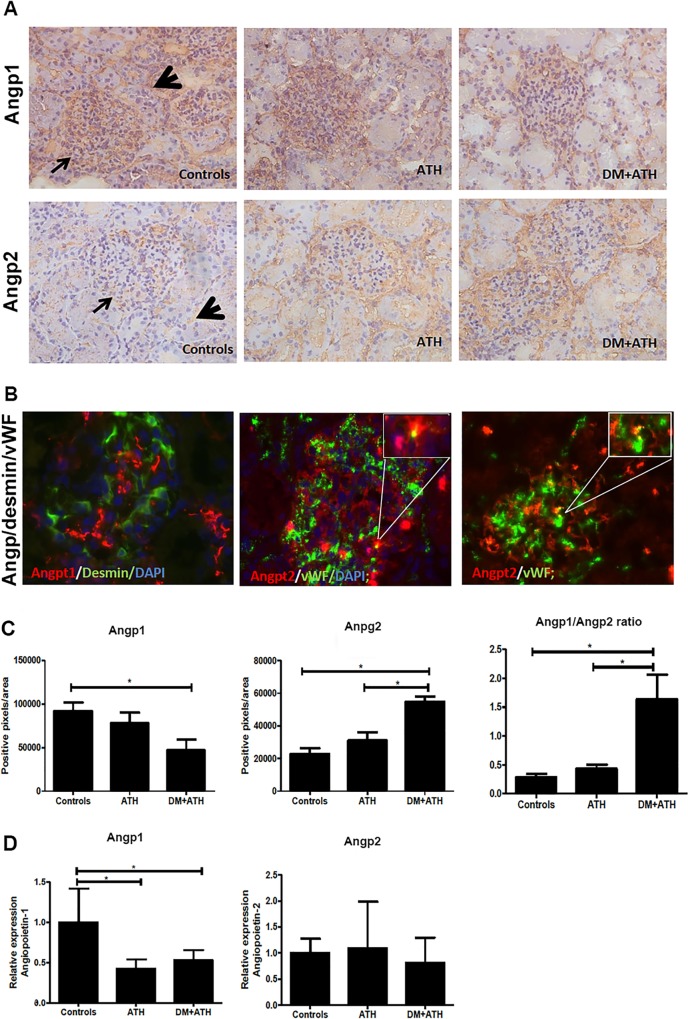
The Angpt2/Angpt1 balance is disturbed in atherogenic DM pigs. A. Representative illustrations of kidney sections stained with Angpt1 (upper panels; arrow: glomerulus; arrowhead: peritubular area) or Angpt2 (lower panels; arrow: glomerulus; arrowhead: tubular staining) in Controls, ATH, and DM+ATH pigs. B. Immunofluorescent double staining of representative kidney sections for desmin (green)/Angpt1 (red;left panel) and vWF (green/Angpt2 (red; middle/right panel). Insets: double positivity for vWF/Angpt2 staining in yellow. C: Quantitative analysis of renal expression of Angpt1, Angpt2 and Angpt2/Angpt1 ratio. D. Relative mRNA expression of Angpt1 and Angpt2. Data are shown as mean ± SEM. *P<0.05 compared to Controls or ATH pigs. Original magnification of A and B: x400.

With regard to Angpt1 staining in the kidneys, we observed positive Angpt1 staining in the glomeruli and in a capillary-like pattern between the tubuli of pigs in the Controls group (Fig 3A). We found no difference in renal Angpt1 protein expression between ATH (78020±12216 pixels/area) and DM+ATH (46763±12360 pixels/area, p>0.05) pigs, but Angpt1expression was significantly higher in the Controls group (91788±9777 pixels/area, p < 0.05; Fig 3A). The Angpt1mRNA levels in the kidney were significantly lower in the DM+ATH and ATH group compared with the Controls pigs (Fig 3D). Double staining of Angpt1 and the pericyte marker desmin revealed predominantly expression of desmin in the glomeruli and Angpt1 expression in the same pattern as described above (Fig 3B; left panel). However, no co-localization was observed, suggesting that other cells than pericytes might produce Angpt1 in the glomerulus.

Angpt2 staining was predominantly present in glomeruli and tubuli (Fig 3A) and showed a significant increase in DM+ATH (54813±3140 pixels/area) pigs compared to the Controls (22862±3354 pixels/area, p<0.001) and ATH (31005±5011 pixels/area, p<0.01) pigs (Fig 3C). Consequently, a higher Angpt2/ Angpt1ratio was observed in DM+ATH (1.63±0.43) pigs compared with Controls (0.28±0.07, p<0.001) and ATH (0.43±0.07, p<0.01) group (Fig 3C). The Angpt2 mRNA levels in the kidney did not significantly differ between groups (Fig 3D). Importantly, additional staining of vWF and Angpt2 revealed co-expression, suggesting that endothelial cells produce Angpt2 (Fig 3B; middle and right panel).

**Fig 4:**
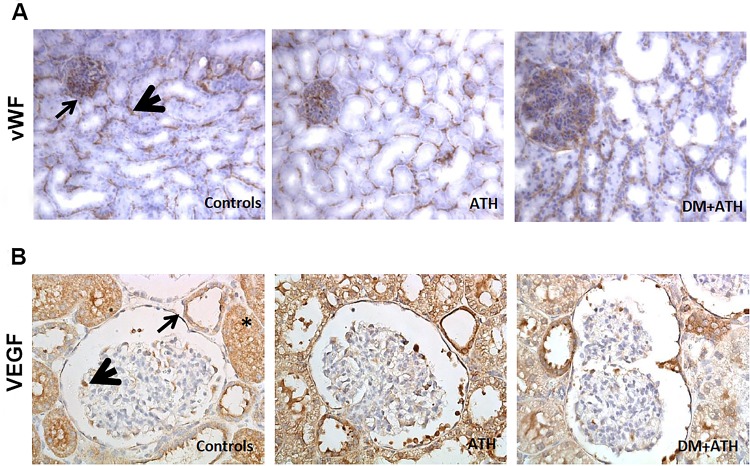
No difference in renal vWf and VEGF-A expression. A. Representative illustrations of kidney sections stained with endothelial marker vWF (arrow: glomerulus; arrowhead: peritubular area) in Controls, ATH, and DM+ATH pigs. B. Representative images of kidney sections stained with VEGF in Controls, ATH, and DM+ATH pigs showing expression in podocytes (arrow head), parietal epithelial cells (thin arrow) and tubuli (asterix). Original magnification of A: x 200 and B: x400.

Staining of the endothelial marker vWF showed expression in the glomeruli and peritubular space, in the same pattern as Angpt1 (Fig 4A). No significant differences were observed in vWF expression between the Controls (86611±6163 pixels/area), ATH (84547±9000 pixels/area) and DM+ATH pigs (102961±13633 pixels/area) (p>0.05).

VEGF-A staining revealed expression in podocytes, parietal epithelial cells and tubuli (most extensively) in all groups (Fig 4B). There was no difference in VEGF-A expression between the DM+ATH (16.94±0.72), ATH (21.22±2.30) and Controls (20.70±1.24) (p≥ 0.05).

### Angpt2/Angpt1 dysbalance and creatinine levels are correlated with capillary tortuosity

**Fig 5:**
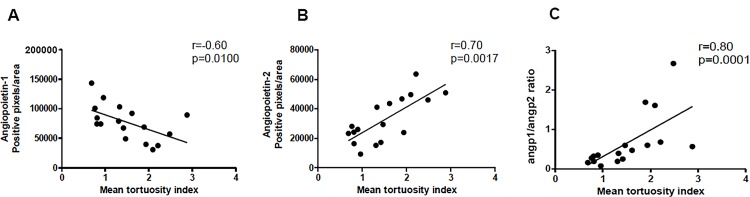
Correlation between capillary tortuosity and Angpt2/Angpt1balance and creatinine levels. Scatter plot showing the correlation of renal protein expression of Angpt1(A), Angpt2 (B), Angpt2/Angpt1ratio (C).

Correlation analyses were performed between renal Angpt1, Angpt2, Angpt2/Angpt1ratio, creatinine levels and albumin/creatinine ratio and oral mucosal capillary tortuosity and density indices. No correlation was found between capillary density index and renal angiopoietin expression or urinary markers for renal function. However, a negative correlation was found between renal Angpt1expression and microvascular tortuosity index (r = -0.60, p = 0.0100; Fig 5A). Moreover, we demonstrated a positive correlation between capillary tortuosity and Angpt2 staining (r = 0.70, p = 0.0017; Fig 5B) and Angpt2/Angpt1ratio (r = 0.80, p = 0.0002; Fig 5C).

No significant correlation was found between urinary albumin/creatinine ratio, creatinine levels and capillary tortuosity index.

## Discussion

In the present study, we show that the combination of an atherogenic diet and recent onset of DM leads to abnormalities in the systemic microvasculature, yielding SDF-detectable increases in capillary tortuosity and increased levels of serum Angpt1. Furthermore, we discovered that glomerular lesions develop during the early stages of DN and that the tight control of the Angpt2/Angpt1balance is perturbed in the kidneys of diabetic atherogenic pigs.

Microvascular dysfunction is one of the most important causes of persistent diabetic complications. This is the result of disturbed hemodynamics and impaired endothelial function induced by metabolic alterations [26,27]. However, the early events involved in the pathogenesis of diabetic microvascular complications are not well understood, and are difficult to study in humans without the use of invasive techniques. SDF imaging allows for the easy, and rapid assessment of the presence of systemic microvascular derangements that could precede the development of diabetic macrovascular complications. In our study, we observed increased capillary tortuosity between the DM+ATH and ATH groups, already at ~15 months following DM induction. Using SDF imaging, we recently demonstrated increased capillary tortuosity in diabetes patients, and reversal of microvascular tortuosity after simultaneous pancreas-kidney transplantation [16]. Moreover, we have previously shown that increased capillary tortuosity in diabetic patients was associated with macrovascular disease [10]. In concordance with our observations, Sasongko et al demonstrated increased tortuosity in patients with diabetic retinopathy, suggesting that this may be an early sign of microvascular damage in DM [28,29]. However, these studies did not include patients with early stage DM and did not focus on the role of angiopoietins in microvascular alterations and renal injury in atherogenic DM.

In addition to increased microvascular tortuosity, we also observed increased serum levels of Angpt-1 in DM+ATH pigs compared with the control group. These data are in line with previous clinical studies in patients with moderate and severe non-proliferative diabetic retinopathy [30] and in patients with hypertension [31] that showed increased Angpt1 serum levels in these patients as well. While serum Angpt1 levels were increased in DM+ATH pigs, we found a decrease in Angpt1 protein and mRNA expression in kidneys of diabetic pigs compared to control groups. This observation suggests shedding of Angpt1 from renal vasculature into the circulation.

Interestingly, DM+ATH pigs were characterized by higher expression levels of Angpt2 protein as compared to the ATH group, resulting in a higher Angpt2/ Angpt1 ratio in the DM+ATH pigs. This is in agreement with Rizkalla and co-workers, who previously have shown that both Angpt1 and Angpt2 protein levels are increased in the early phase of STZ-induced DM in rats [32]. However, disease progression was found to trigger a decrease in Angpt1expression while Angpt2 continued to increase, a switch that both markedly alters but also disturbs the Angpt2/Angpt1balance [32]. Although a difference in angiopoietin expression between rats and pigs cannot be excluded, these findings suggest that angiopoietins undergo time-dependent changes in expression at different stages of DM. Our identification that an increased urinary albumin/creatinine ratio is associated with a higher Angpt2/Angpt1 ratio (in DM+ATH pigs) is in keeping with recent studies that demonstrated an inability to express Angpt1 leads to extensive glomerular damage and proteinuria, indicating a protective role of Angpt1[33]. These data suggest that angiopoietins might play an important role in the pathophysiology of glomerular disease in DN.

Aside from angiopoietins, numerous other mechanisms have been proposed to be causal for microvascular complications in DM, such as VEGF. Under pathological conditions, Angpt2 acts in concert with VEGF to induce inflammatory angiogenesis. By promoting pericyte dropout, Angpt2 will lead to destabilization between ECs and pericytes. In the presence of VEGF, Angpt2 can eventually lead to an active, sprouting state of ECs, but when VEGF is absent, Angpt2 promotes vessel regression [34–36]. Our immunohistochemical assessment of VEGF protein in the different groups revealed high expression levels in podocytes and parietal—and tubular epithelial cells, which is in keeping with literature [34,37–40]. However, in contrast to previous studies, we did not observe differences between the different groups.

An additional observation we made was the marked increase in PAI-1 expression in regions of microvascular injury. PAI-1, a protease and fibrinolysis inhibitor that is poorly expressed in healthy kidneys, which is a critical player in angiogenesis and vascular remodeling [41]. Previous studies in rats and humans by the group of Fogo have shown that the expression level of PAI-1 in normal glomeruli is low and increases in pathological conditions, such as diabetic nephropathy [25,42].

Our observation that glomerular and tubular expression of PAI-1 is increased in DM+ATH pigs is noteworthy due to the fact that: 1) PAI-1 was found to be prominently expressed in Kimmelstiel-Wilson nodules, in particular those with active microvascular damage [25]; 2) unilateral ureteral obstruction [43] and glomerulonephritis [44] have also been associated with increased expression of tubular PAI-1; and 3) endogenous PAI-1 deficiency protects diabetic mice from glomerular injury [45]. Collectively, these data support for a pathogenic role for PAI-1 in diabetic renal disease.

In the current study, there was a tendency towards increased mesangial expansion in the DM+ATH pigs, and significantly more mesangial expansion in kidneys of ATH pigs. The data confirm previous reports on pig studies which revealed that consumption of high-fat diet leads to the development of renal injury, characterized by mesangial expansion and increased plasma creatinine levels [46,47]. Moreover, rodent studies demonstrated that hyperlipidemia exacerbates the development and progression of renal diseases, including DN [48,49]. Furthermore, our study also showed higher creatinine levels in the ATH group than the DM+ATH and Controls, which could be explained by the increased mesangial expansion observed in ATH pigs. The latter has been associated with obliteration of capillary lumen and decreased capillary perfusion in previous studies [50]. Furthermore, our study suggests that the combined hyperglycemia and hyperlipidemia in the DM +ATH pigs, induces a dysbalance in renal angiopoietins that results in albuminuria and endothelial damage in the kidney. The potential contribution of tubular injury to albuminuria needs to be addressed in additional studies.

Some aspects of our porcine study require further discussion. First, it would have been interesting to study an additional group of pigs with only diabetes (i.e. without ATH) to explore the early effects of only hyperglycemia on the microvasculature. However, the clinically most relevant group of DN also reflects DM patients with some presence of atherosclerosis as well. Secondly, it could be argued that the differences in gender, age and weight between healthy control pigs (Controls) versus DM+ATH and ATH pigs may explain part of the difference in the observed capillary tortuosity between these groups. This is, however, unlikely as in a human study, age did not influence capillary tortuosity [16].

In conclusion, we present SDF imaging as a novel means of documenting capillary tortuosity, an event that is illustrative of renal injury in the setting of early DM and atherosclerosis. This technique enables one to non-invasively detect systemic microvascular damage. Furthermore, we also identified changes in the Angpt2/Angpt1 balance may represent initiating events of renal injury in early DM. We propose that the targeted intervention of angiopoietins signaling could be an effective modality in minimizing microvascular damage that could serve as an early initiator of DN.
